# Alteration of Regional Homogeneity within the Sensorimotor Network after Spinal Cord Decompression in Cervical Spondylotic Myelopathy: A Resting-State fMRI Study

**DOI:** 10.1155/2015/647958

**Published:** 2015-10-29

**Authors:** Yongming Tan, Fuqing Zhou, Lin Wu, Zhili Liu, Xianjun Zeng, Honghan Gong, Laichang He

**Affiliations:** ^1^Department of Radiology, The First Affiliated Hospital of Nanchang University, Yong Wai Street, No. 17, Nanchang, Jiangxi 330006, China; ^2^Department of Orthopaedic Surgery, The First Affiliated Hospital of Nanchang University, Yong Wai Street, No. 17, Nanchang, Jiangxi 330006, China

## Abstract

There is a lack of longitudinal research to evaluate the function of neurons' adaptive changes within the sensorimotor network (SMN) following recovery after cervical cord decompression. Regional homogeneity (ReHo) may provide information that is critical to fully understand CSM-related functional neural synchrony alterations. The purpose of this study was to assess the ReHo alterations of resting state-functional MRI (rs-fMRI) within pre- and postdecompression CSM and healthy controls (HC) and its correlations with clinical indices. Predecompression CSM demonstrated a significantly lower ReHo in the left primary sensory cortex and primary motor cortex (PostG/PreG) but enhanced ReHo in the right superior parietal lobule (SPL) compared with HC. In comparison with predecompression CSM, the postdecompression CSM showed increased ReHo in the left PostG/PreG but significantly lower ReHo in the right SPL compared with HC patients. Abnormal ReHo regions in pre- or postdecompression CSM showed no significant correlation with the Japanese Orthopaedic Association (JOA) scores, Neck Disability Index (NDI) scores, and disease duration (*P* > 0.05). This result demonstrated disrupted regional homogeneity within SMN in CSM. This adaptive change in the brain may favor the preservation of sensorimotor networks before and after cervical cord decompression and clinical symptoms independent of ReHo within SMN.

## 1. Introduction

Cervical spondylotic myelopathy (CSM) is a very common cause of spinal cord dysfunction that affects older people by chronic compression of the cervical vertebral column and/or adjacent soft tissue degeneration. The characteristic of CSM is chronic injury of the cervical cord. In some cases, CSM is also a specific incomplete spinal cord injury (SCI).

Currently, most studies of CSM focus on local damage or plasticity of the cervical cord [1, 2]. However, the cortical damage or plasticity in the central nervous system (CNS) may influence the clinical symptoms, manifestations, and functional rehabilitation of CSM. Pathological studies have revealed that apoptotic cell death occurs in axotomized cortical motor neurons in a rat model of SCI [3]. Functional MRI (fMRI), as a powerful noninvasive tool, could be used to probe CSM-related brain structural damage and functional alterations. In a task-state fMRI study, the functional reorganization of sensory and motor cortex was observed, as shown by an increased activation volume in patients with CSM [4]. In patients with CSM, it has been noted that cerebral functional reorganization or plasticity secondary to neuronal damage in the spinal cord is an important pathological mechanism [4–6]. But regional activity synchrony has not been characterized in CSM. Abnormal ReHo may be related to changes in temporal spontaneous neural activity of a certain region. Regional homogeneity (ReHo) reflects the oscillation synchronicity of intraregional cortical neurons (an important aspect of resting-state spontaneous neurons activity), which has been noted as a basic mechanism of neuron function. ReHo may provide information that is critical to fully understanding CSM-related functional brain alterations.

The purpose of this study was to investigate local neural activity alterations within a sensorimotor network (SMN) in CSM by using ReHo at a resting-state. ReHo was measured with Kendall-*w* (Kendall's coefficient of concordance) in regional rs-fMRI time courses, which relied on a voxel-wise analysis of the similarity of the intraregional time series across the whole brain and reflected the temporal synchrony of the regional BOLD signal. Because the SMN is the common and functional-related impairment network in CSM, we chose SMN as an a priori region of interest. Our hypothesis was that CSM would alter or modulate the synchrony of local neural activity within SMN, which is related to the clinical status of the patients. Resting-state fMRI (rs-fMRI) data were acquired from 21 predecompression CSM patients, 21 CSM patients in follow-up 3 months after surgery, and 21 matched healthy controls (HC). ReHo was then compared across pre- or postdecompression CSM and HC and was correlated to disease severity and disease duration to assess its clinical relevance. We hoped to reveal the altered ReHo of local functional connectivity within the SMN and shed light on the possible pathogenesis of CSM following decompression.

## 2. Materials and Methods

### 2.1. Participants

This study was approved by the Medical Research Ethics Committee of the First Affiliated Hospital of Nanchang University. All subjects participated in this study after giving written informed consent. Twenty-one right-handed CSM patients (12 females and 9 males; mean age 47.95 ± 7 years) were recruited at the First Affiliated Hospital of Nanchang University through Convenience Sampling. Twenty-one right-handed age- and sex-matched HC were recruited by advertisements and a convenience sample was conducted at local community. All patients were definitive CSM according to neck MRI, with clinical evidence of CSM. The mean disease duration from disease onset to the date of MRI examination was 14.8 ± 3 months (range 1 month to 9 years). These CSM patients underwent rs-fMRI scan in a follow-up 3 months after surgery. In predecompression CSM patients, the Japanese Orthopaedic Association (JOA) scores and Neck Disability Index (NDI) scores were 11.05 ± 2.52 and 45.1%  ± 12%, respectively. The JOA and NDI scores were 13.55 ± 2.52 and 34%  ± 10%, respectively, in the postdecompression patients. In HC, the JOA and NDI scores were 17 and 5%. HC were screened with a regular neurological examination and presented no history of neurological or psychiatric disorders.

### 2.2. Data Acquisition

Each subject was scanned on a 3.0T MRI scanner (Trio Tim, Siemens, Erlangen, Germany). The subjects were instructed to remain still, keep their eyes closed, and not to think of anything particular during fMRI data acquisition. Functional images were collected axially by using an Echo Planar Imaging (EPI) sequence with the following settings: repetition time (TR)/echo time (TE)/flip angle (FA)/field of view (FOV) = 2,000 ms/40 ms/90°/20 cm, resolution = 64 × 64 matrix, slices = 30, thickness = 4 mm, voxel size = 3.75 × 3.75 × 4 mm^3^, interslice gap = 1.2 mm, and bandwidth = 2,232 Hz/pixel. Two hundred forty rs-fMRI images were acquired. The scan lasted 8.06 minutes.

### 2.3. Data Preprocessing

We used the Data Processing Assistant for Resting-State fMRI Basic Edition (DPARSF) V2.3 (http://www.restfmri.net), running on Matlab 7.8.0 (Mathworks, Natick, MA, USA). Data from the first 10 volumes were discarded to remove the effects of instability of initial magnetic resonance imaging signal and to allow the participants to become used to the environment. The remaining 230 images were preprocessed, which included slice timing, motion correction, coregistration to the structural data, spatial normalization to the MNI (Montreal Neurological Institute) template image, and spatial resampling (3 × 3 × 3 mm). Imaging data for participants with head motion >3 mm in transition or 3° in rotation for the fMRI were discarded. Then, the linear trend was removed. A temporal band-pass filter (0.01–0.10 Hz) was applied to remove very low-frequency drifts and physiological high-frequency noise.

### 2.4. ReHo Analysis

Kendall-*w* was calculated to measure ReHo or the local synchronization of the ranked time series within a functional voxel with the 27 nearest neighboring voxels [7]. For standardization purposes, individual ReHo maps were divided by their global average within the whole-brain mask. Then, spatial smoothing was performed using a 6 mm full-width-half-maximum Gaussian kernel. The procedures used to visualize the ReHo map results were implemented by using the Resting-State fMRI Data Analysis Toolkit V1.8 (http://pub.restfmri.net/) [8].

### 2.5. Statistical Analysis

A second-level random-effect 2-tailed Student's *t*-test (*P* values less than 0.05 were considered significant, corrected with Gaussian random field (GRF) theory multiple comparison correction) was applied to compare the ReHo results between pre- and postdecompression CSM patients and HC within the sensorimotor cortex [9]. A second-level paired 2-tailed Student's *t*-test (*P* values less than 0.05 were considered significant) was applied to compare the ReHo results between pre- and postdecompression CSM patients. The JOA and NDI scores were statistically analyzed by a second-level random-effect 2-tailed Student's *t*-test (*P* values less than 0.05 were considered significant) between CSM and HC, whereas a second-level paired 2-tailed Student's *t*-test was applied to compare the JOA and NDI scores between pre- and postdecompression CSM patients. To perform a correlation analysis between altered ReHo values and clinical measurements, the mean ReHo values of all voxels within the above anomaly regions were separately extracted, and linear regressions were performed to assess the correlations between the ReHo and clinical measures, including the JOA scores, NDI scores, and disease duration (SPSS17.0 software, IBM, 2009).

## 3. Results 

### 3.1. Group Differences of ReHo

**Figure 1:**
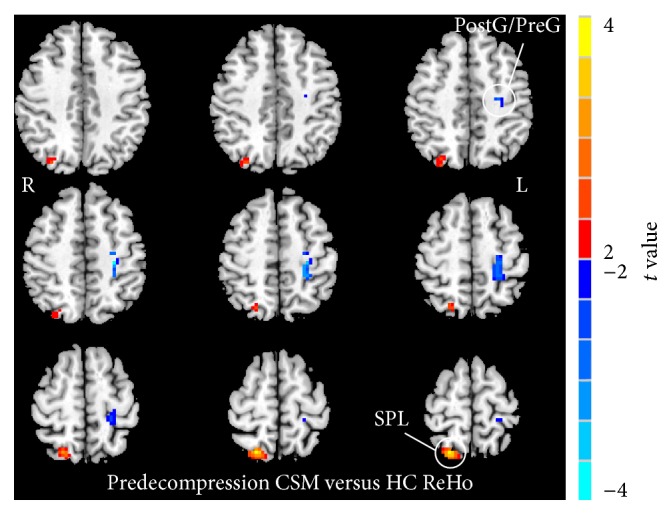
ReHo alterations between the predecompression CSM patients and HC (*P* < 0.05, GRF multiple comparison correction). Yellow and blue colors denote increased and decreased ReHo, respectively. The color bars indicate the *t* values.

Figure 1 shows the group-level ReHo difference within the SMN area between predecompression CSM and HC. Compared with HC, predecompression CSM exhibited significantly lower ReHo (blue spots in Figure 1) in the primary sensory and motor cortices (a joint cluster in postcentral gyrus and precentral gyrus, PostG/PreG). However, predecompression CSM showed a significantly higher ReHo (red spots in Figure 1) in the sensory association cortex (superior parietal lobule, SPL). The *t* value and cluster size of predecompression CSM versus HC ReHo differences are listed in Table 1.

**Figure 2:**
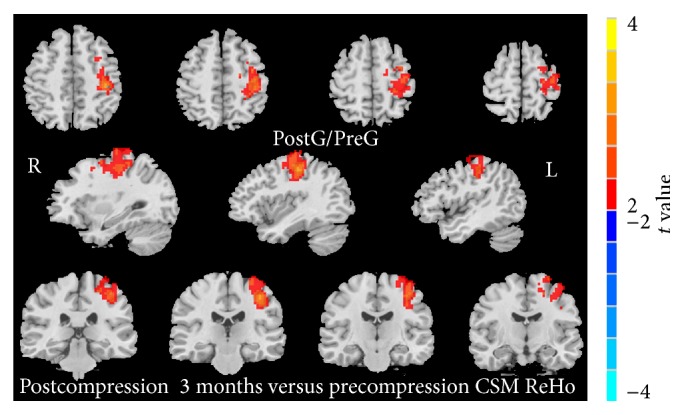
Postcompression versus precompression CSM patient ReHo difference (paired 2-tailed *t*-test, *P* < 0.05, GRF multiple comparison correction).

**Figure 3:**
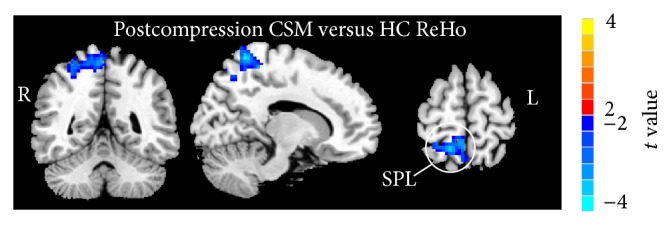
Postcompression CSM versus HC ReHo difference (*P* < 0.05, GRF multiple comparison correction).

Three months after surgical spinal cord decompression, the postdecompression patients showed increased ReHo in the left PostG/PreG compared with predecompression (red spots in Figure 2). Compared with HC, postdecompression CSM patients demonstrated a significantly lower ReHo in the right SPL (blue spots in Figure 3).

### 3.2. ReHo Correlated with Clinical Indicators

The JOA and NDI scores of pre- and postdecompression CSM and HC exhibited significant differences (*P* < 0.05).

Then, linear regressions were performed to assess the associations of ReHo to distinct clinical measures, including the JOA scores, NDI scores, and disease duration. However, no brain region demonstrated statiscal correlations with the JOA scores, NDI scores, and disease duration in the predecompression (Table 2) or predecompression CSM group (*P* > 0.05) (Table 3).

## 4. Discussion

ReHo reflects the resting-state neuronal synchronization of intraregional activities [7]. ReHo's Kendall-*w* coefficient analyzes the individual element via a calculation that involves the synchronization of the functional voxels and its neighboring voxels, which reflects the local brain blood oxygen level at the time of synchronization. ReHo abnormalities may reflect abnormal local neuronal activity. A lower ReHo may represent local intraregional reduced neuron activity or regular disorder [7], which can strongly affect the brain neurons involved in information processing, as demonstrated in acquired deafness [10] and severe depression [11]. Enhanced ReHo reflects neuron activity in the region of the brain by increased time synchronization and strengthened brain function [12].

The results of decreased ReHo in the left M1/S1 (left PostG/PreG) in predecompression CSM are consistent with other functional neuroimaging studies [13, 14] and similar to previous studies in SCI [15–18]. Normally, the M1 is involved in the planning, control, and execution of voluntary movements through the spinal cord to muscles. S1 is a site for the integration of inputs from different afferent sources, which lead to perceptual recognition of the presence, location, intensity, submodality and quality of touch, innocuous thermal sensibility, and pain [19]. Abnormal N-acetylaspartate/creatine in the motor cortex, as disclosed by proton magnetic resonance spectroscopy in CSM, was indicative of neuronal damage or dysfunction [6]. Axon damage can be retrograde or anterograde damage to the nucleus, which has led to speculation that metabolism or function of nerve nuclei and central cortex may change and lose gray matter M1 volume [20]. In this study, one explanation for the reduced ReHo of M1/S1 is the injury of the cortical neurons. Another explanation is injury of the corticospinal tracts, including incoming or outgoing fibers of M1/S1. Regional brain synchrony is lost in response to abnormal cortical projection (reflex pathways) from the cervical cord or thalamus in CSM patients. In a SCI study, similar findings also support our finding, such as decreased FA (which is indicative of fiber loss and/or demyelination) in the S1 and corticospinal/corticopontine tracts [21]. This study found that ReHo decreased in the left M1/S1 before decompression, which indicates that patients with CSM have functional damage of the sensorimotor cortical area.

Three months following surgical cervical cord decompression, the postdecompression CSM patients showed increased ReHo in the left M1/S1 compared with predecompression CSM patients. Compared with HC, the postdecompression CSM patients exhibited cortex synchronicity that was restored to the normal level and reduced preoperative cortical reorganization. After decompression, the cervical cord fibers' lateral growth [21, 22], which built new synapses within sensorimotor cortex or removed original inhibitory synapses [18, 23, 24], may explain this phenomenon. Primary sensorimotor cortex neurons synchronicity change in pre- and postdecompression CSM confirmed that the sensorimotor cortex reorganization participates in the function recovery of postdecompression CSM patients.

In the current study, ReHo was significantly increased in the right SPL in predecompressed CSM patients, which reflects the enhancement of the local synchronization of spontaneous neural activities in this region. The SPL is a secondary sensory cortex, is involved with spatial orientation, and receives a great deal of both visual and sensory input from one's hand [25]. The damage of SPL can cause contralateral astereognosis, ipsilateral spatial neglect, visual related movement disorders, and operation processing spatial orientation [26], among other symptoms. The SPL round trip fiber contact with M1/S1 bears the advanced integration functions, feeling, and other information from various brain regions in the final comprehensive analysis and judgment. Increased ReHo of SPL in predecompression CSM may involve functional integration and/or regulation in the bulk of the injury information from the spinal cord, thalamus, or primary sensory cortex [27]. Secondary sensory cortex ReHo compensatory rising on predecompression CSM may explain why the clinical signs and symptoms of CSM are not directly consistent with the degree of spinal cord compression.

Postdecompression CSM patients demonstrated a significantly decreased ReHo in the right SPL compared with HC. On the one hand, the possible reason for this decrease is that there was decreased stimulation input information from a cervical cord injury and M1/S1. The compensatory neuromodulation power between SPL and M1/S1 becomes lower immediately following decompression surgery. On the other hand, another possible reason is that although the stimulation information input decreased, the SPL reorganization in predecompression patients remained an efficient way to process information at 3 months following decompression. That is, SPL's neurons had not yet returned to a normal activity level, which shows the consistent efficiency of neurons when dealing with normal sensory information. Thus, SPL showed decreased ReHo when dealing with normal sensory information compared with HC. Whether abnormal ReHo returns to normal after a longer follow-up needs further research. Three months after surgical spinal cord decompression, the brain cortex of SPL appears to be reorganized such that the cortex assigns more elaborate functional regional segregation and integrates low-energy and high-efficiency information processing in postdecompression CSM patients; in the cortex, horizontal intracortical axons and dendrites are interconnected by different sensory representations of the sensory cortex and thus support this idea [23].

However, no significant correlations were observed in the cortical ReHo among the SMN and JOA scores, NDI scores, and disease duration, which suggested that the functional change of CSM patients is primarily caused by local damage in the spinal cord, not cortex injury. First, as for secondary destructive mechanisms, the spinal cord has an innate ability to recover varying degrees of sensory, autonomic, and motor function and plasticity of the human brain [28]. This interplay between the ongoing destructive mechanisms and the innate reparative processes eventually reaches a balance that is often described in terms of adaptive or maladaptive plasticity. Second, in brain studies of CSM patients, no direct correlation was observed between the JOA score and motor-related activation [4] or neuronal metabolite ratios [6]. Third, although the JOA system is recommended, it presents some clinical disadvantages, such as the sensitivity, effectiveness, and ignorance of its physical functions for the cervical spine (e.g., range of motion of the neck, pain) [29]. For the JOA and NDI scores obtained using the questionnaire method, a patient may have problems such as subjective consciousness and a lack of objectivity [30].

There is little documented evidence for lateralization plasticity in the human brain. Decreased/increased ReHo in CSM patients only occurs on one side. On the one hand, some CSM patients show asymmetry compression or injury of the cervical cord. Sensory or motor impairment was present with more severe relatively on one side in some CSM patients, as was lateralization of the spinal cord compression. There is considerable asymmetrical plasticity, whereas the degrees and extent of neuroplasticity depend on multiple factors, including the level and extent of compression. Some studies have concluded that somatotopic organization is left unchanged following SCI recovery, but movements of muscle groups rostral to the site of injury result in increased activity in the primary motor cortex and associated regions of the cerebellum [31]. On the other hand, the dominant hemisphere of the brain may play a role.

## 5. Limitations

Our data reveal only cortical reorganization, which may be different with the degree of compression at different levels within the spinal cord. The next step is combining it with cervical spinal injury, as revealed by DTI and cortex injury. Different degrees or levels of cervical cord compression may lead to different degrees of brain cortex reorganization and compensation, a lack of analysis of the correlation between the degree of cervical cord injury or different cervical levels and the degree of sensory motor cortex reorganization. However, because CSM decompression adopts a mental internal fixation, it is difficult to determine postdecompression cervical spinal DTI damage scanning artifacts, perform a correlation analysis of postdecompression cervical cord structure recovery, and undergo cortex reorganization. In addition, this study had only a 3-month follow-up after decompression. Thus, a longer follow-up may need to be considered.

## 6. Conclusions 

In summary, our findings provide further evidence of disrupted regional homogeneity within the sensorimotor network in CSM patients. Adaptive changes in the brain may favor the preservation of cortical sensorimotor networks before and after cervical cord decompression and clinical symptoms independent of ReHo within SMN.

## Figures and Tables

**Table 1:** Significant ReHo differences between predecompression CSM patients and HC subjects (*P* < 0.05, corrected for multiple comparisons).

Functional area	Brain regions	BA	Peak location (MNI)			Number of voxels	Peak intensity value
			*x*	*y*	*z*		
CSM patients >HC							
Sensory association cortex	Right SPL	7	21	−66	66	87	3.829
CSM patients <HC							
Primary sensory/motor cortex	Left PostG/PreG	1, 2, 3, 4	−27	−27	51	89	−3.894

Notes: BA, Brodmann area; MNI, Montreal Neurological Institute; SPL, superior parietal lobule; PostG, postcentral gyrus; PreG, precentral gyrus.

**Table 2:** Correlation between clinical status indexes and mean ReHo values in predecompression CSM patients.

	Correlation coefficient (*P* value)		
	JOA scores	NDI scores	Disease duration
Right SPL	−0.212 (0.357)	0.243 (0.289)	0.135 (0.560)
Left PostG/PreG	−0.037 (0.874)	0.124 (0.591)	0.244 (0.286)

**Table 3:** Correlation between clinical status indexes and mean ReHo values in postdecompression CSM patients.

	Correlation coefficient (*P* value)		
	JOA scores	NDI scores	Disease duration
Right SPL	−0.202 (0.347)	0.145 (0.341)	0.132 (0.410)
